# Sensitive detection of copper ions via ion-responsive fluorescence quenching of engineered porous silicon nanoparticles

**DOI:** 10.1038/srep35565

**Published:** 2016-10-18

**Authors:** Jangsun Hwang, Mintai P. Hwang, Moonhyun Choi, Youngmin Seo, Yeonho Jo, Jaewoo Son, Jinkee Hong, Jonghoon Choi

**Affiliations:** 1 School of Integrative Engineering, Chung-Ang University, Seoul 06974, Republic of Korea; 2 Department of Bioengineering, Swanson School of Engineering, University of Pittsburgh, Pittsburgh, PA 15261 USA; 3 School of Chemical Engineering & Materials Science, Chung-Ang University, Seoul 06974, Republic of Korea

## Abstract

Heavy metal pollution has been a problem since the advent of modern transportation, which despite efforts to curb emissions, continues to play a critical role in environmental pollution. Copper ions (Cu^2+^), in particular, are one of the more prevalent metals that have widespread detrimental ramifications. From this perspective, a simple and inexpensive method of detecting Cu^2+^ at the micromolar level would be highly desirable. In this study, we use porous silicon nanoparticles (NPs), obtained via anodic etching of Si wafers, as a basis for undecylenic acid (UDA)- or acrylic acid (AA)-mediated hydrosilylation. The resulting alkyl-terminated porous silicon nanoparticles (APS NPs) have enhanced fluorescence stability and intensity, and importantly, exhibit [Cu^2+^]-dependent quenching of fluorescence. After determining various aqueous sensing conditions for Cu^2+^, we demonstrate the use of APS NPs in two separate applications – a standard well-based paper kit and a portable layer-by-layer stick kit. Collectively, we demonstrate the potential of APS NPs in sensors for the effective detection of Cu^2+^.

Copper is an essential trace element in most plants, cells, and animals, whereby fundamental physiological processes require the participation of Cu ions. The production of skin and hair pigments as well as the development of bone, for example, all make use of Cu ions, which participate in the utilization of iron and zinc^1^. Not surprisingly, copper deficiency or the over-accumulation of copper in the human body can cause various disorders such as Wilson’s disease^2^ or Menkes disease^3^. In particular, high levels of Cu have been shown to enter an agricultural system through animal manure or litter present on farmlands^4^. Such Cu accumulation is severely toxic to plants and some bacteria, and ultimately affect humans through the food chain^5^. In addition to agriculturally-derived Cu pollution, transportation-mediated emissions also contribute to the production of heavy metal particulates such as Cu, which settle and deposit at the bottom of rivers and lakes^6^,^7^. According to the World Health Organization (WHO), the maximum permissible limit of Cu(II) in drinking water is 1.0 mg/L^8^. The standard method of measuring copper ions, however, employs ion-selective electrodes, which presents difficulties when considering the presence of various non-specific biomolecules (*i.e.* proteins, enzymes, other ions). From this perspective, a simple and convenient method to detect Cu ions with high specificity and sensitivity is desirable.

Various functionalized nanoparticles have been examined for their ability to detect specific metal ions^9^,^10^,^11^,^12^. In particular, monocrystalline silicon wafers – used in chemical sensors^13^, energy harvesting^14^, and solar cells^15^ – are used to generate porous silicon nanoparticles (PS NPs). Given the size-dependent tunable photoluminescence (PL), high quantum yield, broad excitation spectra, low photobleaching, and low toxicity of PS NPs, they thereby present a viable means to increasing the sensitivity of Cu detection^16^,^17^,^18^. Various methods have been reported for the synthesis of such PS NPs^9^,^10^,^11^,^12^,^13^,^14^,^15^, including their fabrication from Si wafers via electrochemical wet etching^19^ as previously demonstrated by our group. Interestingly, previous studies on PS NPs report that oxide-terminated silicon NPs often experience PL-quenching under a variety of conditions^20^,^21^,^22^, in which Cu^2+^ can dramatically reduce the quantum efficiency of porous silicon by capturing excited carriers and interrupting radiative recombination processes^23^,^24^. In particular, the fluorescence of alkyl-terminated PS NPs (APS NPs) exhibit a linear relationship with the concentration of Cu ions, in which bare PS NPs do not show significant PL-quenching in the presence of Cu ions^23^,^25^.

In this study, we demonstrate an efficient method to prepare surface-modified photoluminescent APS NPs from Si wafers to obtain an extremely bright and highly stable PL (Fig. 1). Specifically, freshly-prepared PS NPs are grafted with undecylenic acid (UDA) or acrylic acid (AA) and evaluated for their potential to improve Cu^2+^ sensitivity and optical properties. Various Cu sensing conditions, such as pH, duration, and temperature are then evaluated for the APS NPs. Finally, we demonstrate the use of APS NPs in two separate applications: 1) a well-based paper kit for use in the lab and 2) a portable stick kit for potential on-site use. The paper kit consists of hydrophilic sections covered with APS NPs and hydrophobic barriers prepared by wax printing and thermal treatment. The portable stick kit, on the other hand, is based on a layer-by-layer (LbL) multilayering method. Put together, we engineer APS NPs to selectively exhibit PL quenching in the presence of Cu^2+^ and demonstrate their potential use in a variety of Cu sensors.

## Results and Discussion

### Preparation of Porous Si NPs

**Figure 2:**
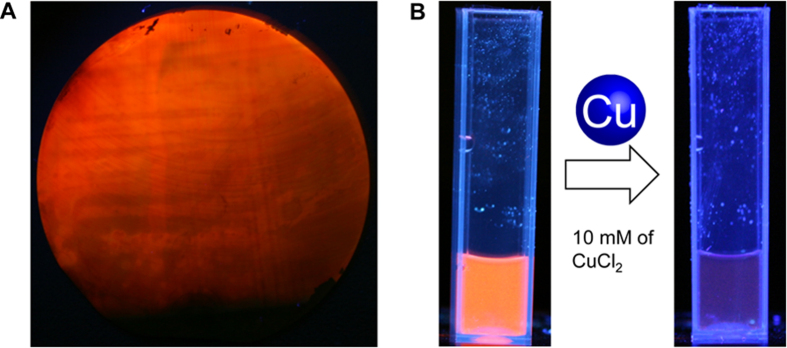
Preparation of Photoluminescent PS NPs. (**A**) Si wafers are electrochemically etched for 30 minutes under a current of 260 mA and an acid injection rate of 5 mL/s. After washing in excess deionized water and methanol, the wafers are dried with N_2_ (g) and exhibit fluorescence at approximately 640 nm under UV (365 nm excitation). (**B**) APS NPs are collected via wafer sonication in ethyl alcohol and emit an orange-red fluorescence under UV, which is quenched upon the addition of 10 mM of CuCl_2_.

**Figure 3:**
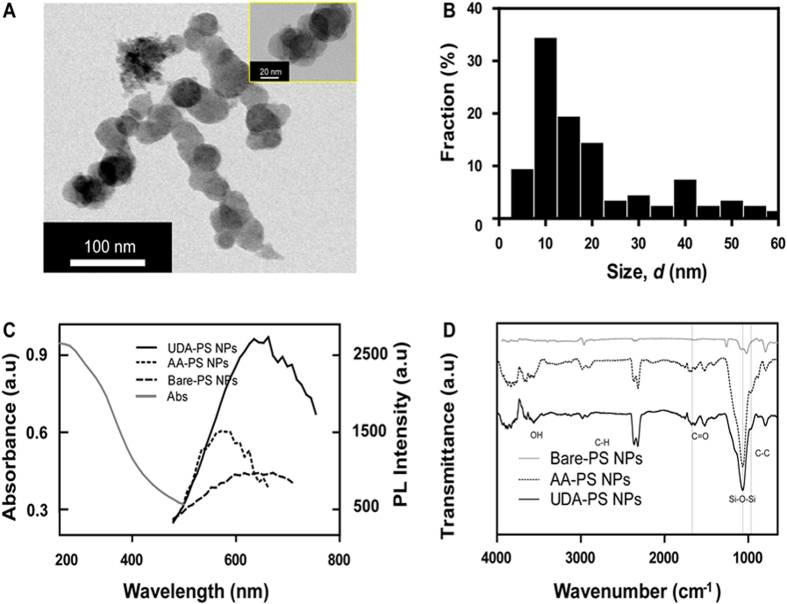
Characterization of PS and APS NPs. (**A**) TEM image of PS NPs obtained after the sonication of etched Si wafers. High-resolution TEM image (inset) shows the crystalline structure of Si nanoparticles. (**B**) Image analysis of TEM images, summarizing the size distribution of a polydisperse nanoparticle suspension in water (*n* = 150). (**C**) Photoluminescence curves of PS and APS NPs indicate an increased level of PL intensity upon hydrosilylation. (**D**) FTIR analysis of PS and APS NPs shows the appearance of Si–C and Si–O peaks after hydrosilylation.

Porous Si NPs (PS NPs) are prepared from electrochemically-etched Si wafers. Typical etching of a 500 mm As-doped wafer is carried out laterally for 1 hr in an electrochemical cell at an optimized pumping speed of 5 mL/min to ensure treatment of the entire surface. Freshly prepared etching solution (HF–H_2_O–ethyl-alcohol) is used to stabilize the current flow in the cell, typically maintained at 120–250 mV. Following the etching procedure, the wafers are washed with excess deionized water and methanol and dried with N_2_ (g). The resulting etched porous Si surface emits an orange-red photoluminescence under ultraviolet (UV) excitation at 365 nm (Fig. 2A). PS NPs, obtained by sonicating the dried wafers for 90 minutes in ethyl alcohol, exhibit an orange-red photoluminescence that is quenched in the presence of Cu^2+^ ions (Fig. 2B). Transmission electron microscope (TEM) images of the PS NPs show the crystalline structure, size, and shape of the NPs; while individual monocrystals are not found among the NPs, Si nanocrystalline domains trapped in larger pieces of the porous silicon structure are observed (Supporting Fig. S2A,B). The PS NPs have an average diameter ranging from 20–80 nm as assessed via TEM image analysis (Fig. 3A).

### Characterization of Alkyl-Terminated PS NPs

PS NPs are reacted with either UDA or AA to generate alkyl-terminated PS NPs (APS NPs). Briefly, UDA-PS NPs are produced by heating an ethanol solution of PS NPs (10 wt% UDA) to 95 °C for 16 hours under N_2_. Hydrosilylation of the NPs is observed when the turbid solution becomes transparent. AA-PS NPs are produced by bubbling an ethanol solution of PS NPs (10 wt% AA) with N_2_ to remove any dissolved oxygen that could inhibit the radical-initiated reaction. The PS NP suspension is then placed in a photochemical reactor and run for 24 hours at 40 °C until a clear suspension is obtained. ASP NPs, obtained after centrifugation and multiple washing steps in ethanol, are resuspended in deionized water for subsequent experiments.

The average diameter of the APS NPs is 45 nm as assessed via TEM image analysis (Fig. 3B). The absorption spectrum of PS NPs shows a maximum peak at 260 nm (Fig. 3C, gray line). Upon excitation, the PL intensity of AA-PS NPs is at least twice as strong as that of bare PS NPs. The PL intensity of UDA-PS NPs is even stronger, with a 10 fold increase in intensity over that of AA-PS NPs. Both AA-PS NPs and UDA-PS NPs exhibit maximum PL intensity at approximately 650 nm and corroborate the rearrangement of surface structure and subsequent promotion of efficient proton emission^19^; the increased PL of alkyl-passivated PS NPs is most likely due to the quantum confinement effect and defect localization at the Si–SiO_2_ interface^26^. Furthermore, the quantum yield (Ф) of UDA-PS NPs is 0.11 when indirectly compared to the value of Rhodamine (Supporting Fig. 3). Aside from a change in PL intensity, we also observe a negative increase in the zeta potential of the NPs after hydrosilylation; bare PS NPs and APS NPs have a surface charge of −25 ± 2 mV and −85 ± 3 mV, respectively (Supporting Fig. S4A). This increase in negative surface charge notably improves the dispersity of APS NPs and ensures their stable PL in acidic solutions. Finally, the surface composition of the APS NPs, created either via photo- or thermal-initiated hydrosilylation, is analyzed by Fourier transform infrared (FTIR) spectroscopy (Fig. 3D). The ASP NPs display distinctive features of surface alkyl groups (CH_2_: 2939, 2866, and 1498 cm^−1^; C-C: 869 cm^−1^) and strong vibrational features of SiO_*x*_ peaks (1050 cm^−1^). The appearance of a carboxyl band at 1717 cm^−1^ further confirms the presence of UDA or AA molecules on the surface of the NPs.

### Stability and Optical Property of APS NPs

**Figure 4:**
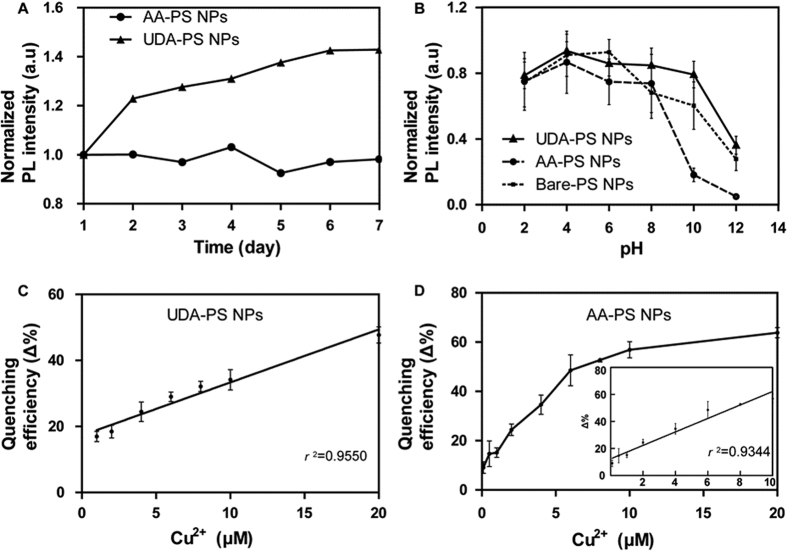
APS NP Stability and [Cu^2+^]-dependent PL Quenching (Δ%). (**A**) Variations in PL intensity are measured for 7 days for UDA-PS NPs and AA-PS NPs in water, in which a continuous increase in intensity is observed for UDA-PS NPs. (**B**) The effect of pH on PL intensity is measured for UDA-PS NPs and AA-PS NPs. While all three NPs show similar PL intensities in acidic solutions, UDA-PS NPs retain the most intensity in basic solutions. (**C**) UDA-PS NPs and (**D**) AA-PS NPs are quenched by Cu^2+^ in a concentration-dependent linear manner between [Cu^2+^] of 1–20 μM and 1–10 μM, respectively. The quenching efficiency is the average of 10 individual measurements, with the maximum and minimum values excluded.

The fluorescence stability of APS NPs is analyzed by continuously measuring the PL intensity for 7 days (Fig. 4A). The AA-PS NPs show constant PL intensity in aqueous solution, while the UDA-PS NPs show increasing PL intensity over 7 days. Long hydrocarbon chains terminated on bare PS NP surface would deter direct contacts between Si nanocrystals and oxygen molecules leading to a quenching^23^. When free radicals in solutions interact with the surface of PS-NPs, hydrocarbon chain on particles let Si core be free from their interference while free radicals attack Si-C bonds instead. It may result in stable and increased PL intensity from UDA-PS NPs as we observed. In addition, it should be noted that variations in pH of the solution would affect the PL intensity of the NPs, as shown in the measurement of PL intensities from APS NP samples depending on the solution pH (Fig. 4B). Specifically, while the PL intensity of the AA-PS NPs remains stable in an acidic environment, it significantly decreases when the pH is over 8.0 and exhibiting complete quenching of PL in an alkaline solution. The bare PS NPs, on the other hand, maintains stable PL intensity when the pH < 10.0. The UDA-PS NPs show stable PL intensity over a broad range of pH values spanning from acidic to basic conditions, most likely due to the UDA molecules on the NP surface may reduce the rate of degradation.

### Detection of Cu^2+^ in Solution

To determine the capacity of APS NPs for the detection of copper ions, we evaluate the relationship between PL intensity and copper ion concentration (Fig. 4C,D). At a copper concentration of 1 M, PL quenching efficiency (Δ%) is approximately 55% for UDA-PS NPs and 83% for AA-PS NPs; the quenching reaction is complete within 30 s in water (Supporting Fig. 5). Here, quenching efficiency (Δ%) represents the extent of copper ion-mediated reduction in PL intensity (Eq. 1), in which an increase in Δ% represents the presence of higher levels of copper ion. For UDA-PS NPs, PL quenching efficiency increases linearly with an *r*^2^ value of 0.9550 for [Cu^2+^] between 1–20 μM (Fig. 4C); the quenching efficiency is 18% for a 1 μM Cu^2+^ solution, which is the minimum measurable amount of copper ions, and reaches a maximum measurable amount of 55% at 20 μM, beyond which PL quenching is maximized. On the other hand, PL quenching of AA-PS NPs increases linearly with an *r*^2^ value of 0.9344 for [Cu^2+^] between 1–10 μM (Fig. 4D). In particular, 60% quenching efficiency is measured for AA-PS NPs in 10 μM of copper ions, thereby suggesting their higher sensitivity relative to UDA-PS NPs. PL quenching begins to plateau at 20 μM and saturates at 1 M of copper ions. Finally, the capacity of UDA-PS NPs to exhibit fluorescent quenching is examined for other transition metal ions and alkali/alkaline earth metal ions, including Fe^2+^, Na^+^, K^+^, Mg^2+^, Mn^2+,^ Ca^2+^, and Cu^2+^ (Supporting Fig. 1). The UDA-PS NPs show quenching efficiencies between 3% and 20%, depending on the type of ion, of which Fe^2+^ ions show a particularly high quenching efficiency of 40., It should be noted that other copper ion sensing NPs, such as carbon dots^22^,^23^,^24^,^25^, also demonstrate a strong selectivity of fluorescence quenching by copper and other positive ions (i.e. Fe^2+^); the complexation of NPs with organic molecules specific for copper ions^25^ (e.g. N-(2-aminoethyl)-N,N,N′tris(pyridin-2ylmethyl)ethane-1, 2-diamine) may act to further increase the selectivity of our platform. These results collectively corroborate previous results that demonstrate copper ion-specific fluorescence quenching of alkyl-functionalized PS NPs, in which the capture of excited carriers and the interruption of radiative recombination processes provide the driving force behind PL quenching.

### Detection of Cu^2+^ with a Well-based Paper Kit

**Figure 5:**
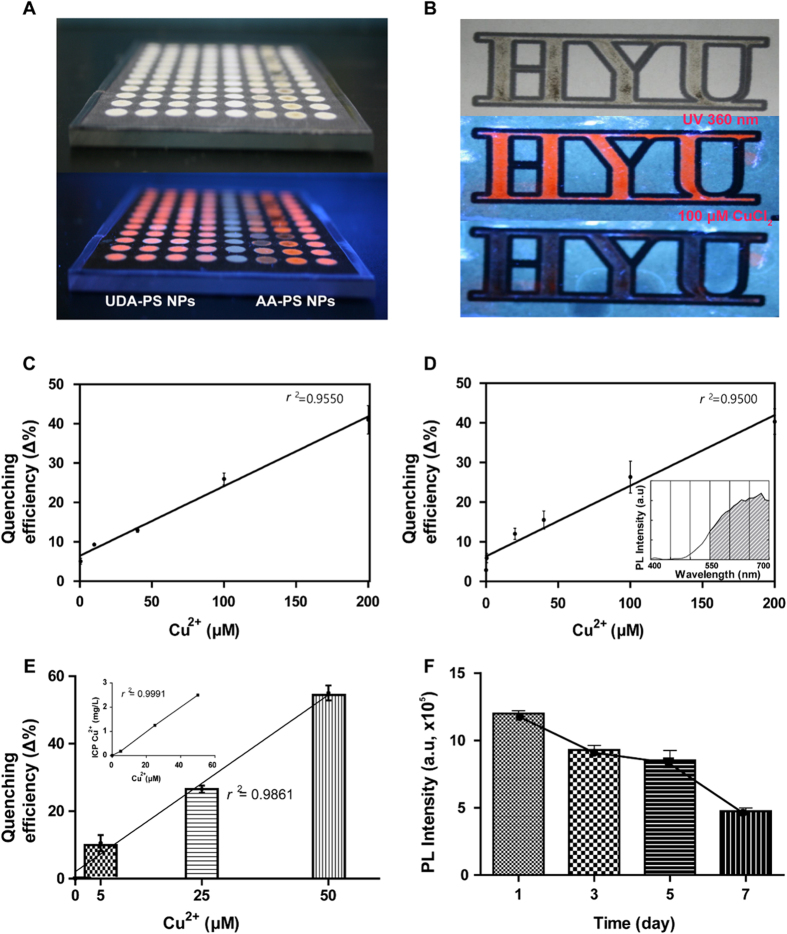
Evaluation of 96-well Paper Kit Functionalized with APS NPs. [Cu^2+^] is assessed via quenching ratio in a paper kit functionalized with (**A**) UDA-PS NPs or AA-PS NPs on a 96-well paper kit is prepared using a wax printer, in which wax is used to construct hydrophobic walls (after baking the printed paper at 130 °C for 3 min) for 96 wells (6 mm in diameter). (**B**) A custom designed letters coated with APS NPs: (top) bright-field image, (middle) fluorescent image before the addition of 100 μM CuCl_2_, (bottom) fluorescent image after the addition of 100 μM CuCl_2_. (**C**) The detected copper-ion concentration was measured by the quenching ratio in a paper kit functionalized with UDA-PS NPs. (**D**) The detected copper-ion concentration was measured by the quenching ratio in a paper kit functionalized with AA- PS NPs (The inset shows the calculated integrated PL intensity, excluding data beyond the wavelength range of visible light.) (**E**) A linear relationship is observed between the fluorescence quenching percentage and [Cu^2+^] in tap water (*n* = 3); the inset shows the actual amount of copper ions as assessed via ICP mass spectrometry. (**F**) The fluorescence stability of the paper kit decreases as a function of time; fluorescence intensity values are integrated under the emission spectrum curve from 550 to 700 nm.

APS NPs are used in conjunction with a 96-well-based template, which consists of a coating of APS NPs in each well surrounded by a hydrophobic wax barrier (Supporting Fig. S2C,D). Various concentrations of copper ions are then released into the wells to evaluate the Cu^2+^ detection capacity of APS NPs in the context of a paper kit. Specifically, 10 μL of Cu^2+^ aqueous solutions with concentrations ranging from 1 to 200 μM are added into individual wells, dried, and measured for PL intensity (Fig. 5A). The potential of an APS NP-based paper kit is further corroborated via a custom design (Fig. 5B), which shows the quenching of PL intensity upon addition 100 μM of Cu^2+^. Upon further examination of the paper kit, we observe that the quenching efficiency depends on the following: the location of sample loading, extent of paper wetting, and the presence of dust, artifacts, proteins, or substrate papers, and types of solution that could interfere with the blue emission of APS NPs (Supporting Fig. S4B,C). To minimize non-specific interferences, we select PL emission wavelengths from 550 to 700 nm for both AA-PS NPs and UDA-PS NPs (Fig. 5D, inset). The paper kit prepared from UDA-PS NPs can detect a minimum Cu^2+^ concentration of 0.5 μM with a corresponding PL quenching of 7%. Increasing the concentration of Cu^2+^ further enhances the PL intensity in a linear fashion for concentrations up to 200 μM, with an *r*^2^ value of 0.9550 (Fig. 5C). Similarly, the paper kit coated with AA-PS NPs detects a minimum Cu^2+^ concentration of 0.1 μM with a corresponding PL quenching of 7%. The PL intensity also shows a linear range of detection, with a high *r*^2^ value of 0.9500 for concentrations up to 200 μM (Fig. 5D). Finally, the capability of the assay for the accurate detection of copper ions in tap water is assessed via the addition of 5, 25, or 50 μM of CuCl_2_ in tap water to the paper kit (Fig. 5E). We observe a quenching percentage of 10 ± 2.3% upon the addition of 5 μM CuCl_2_ and a linear relationship (*r*^2^ value of 0.9861) between [Cu^2+^] and fluorescence quenching (Δ%). Additionally, the actual copper ion concentration in the samples are assessed via inductively coupled plasma (ICP) mass spectrometry (Fig. 5E, inset), and show comparable values to the theoretical amount of copper (Table 1). The limit of detection of the assay is 5 μM of copper ions.

It should be noted, however, that compared to the results obtained in solution (Fig. 4), the PL quenching is not significantly high on the paper kits, which could be explained by the fewer number of APS NPs in the paper kit and the limited reaction between the Cu^2+^ and the NPs. Furthermore, when testing the paper kit with a biologically-relevant sample containing bovine serum albumin, we observe a deterioration in the fluorescence stability due to protein aggregation with the NPs (Supporting Fig. 5C). Finally, the fluorescence stability of the paper kit is monitored under aerobic conditions in a dark room at room temperature, in which a reduction in PL intensity is observed after 3 days (Fig. 5D). Both non-radiating and radiating recombinations may have affected the fluorescence intensities of the spots over time. In addition, the interaction of oxygen with the fluorescent spots would also have decreased the PL intensities.

### Detection of Cu^2+^ with a Portable Stick Kit

**Figure 6:**
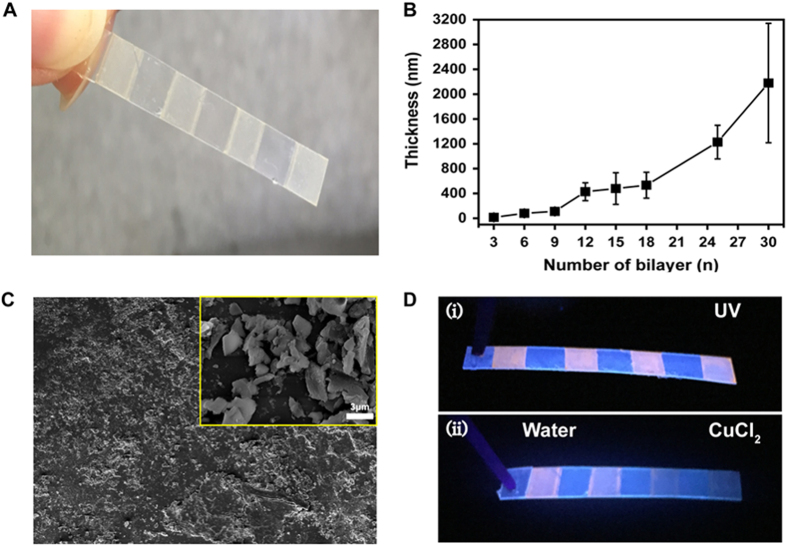
Portable APS NP-based Flexible Stick Kit. (**A**) Photograph of (bPEI/APS NP)_30_ LbL film on a PDMS-patterned flexible substrate. (**B**) The thickness of the (bPEI/APS NP) LbL film shows an exponential increase with increasing iterations. (**C**) FE-SEM image of (bPEI/APS NP)_30_ LbL film; inset shows a magnified micrograph of the surface. (**D**) (i) Patterned (bPEI/UDA)_30_ stick displaying orange-red fluorescence under UV excitation; (ii) water is dropped onto the two films on the left while 10 mM of CuCl_2_ solution is dropped onto the two films on the right.

We prepare a flexible stick coated with a Cu^2+^-responsive fluorescent film via LbL (Fig. 6A). Briefly, branched polyethylenimine (bPEI) is layered onto a PDMS-patterned OHP substrate, after which UDA-PS NPs are layered on top in a LbL assembly process. Using ethanol as a solvent, we assemble both bPEI and APS NPs utilizing hydrogen bonding between –COOH of the APS NPs and –NH_2_ of bPEI. We observe an exponential increase in the film thickness with the number of bilayers (Fig. 6B), demonstrating the successful alternating deposition of APS NPs and bPEI. The surface morphology of the multilayer films – observed via field-emission scanning electron microscopy (FE-SEM) – is rough, partially due to the aggregation of APS NPs with bPEI during the process (Fig. 6C). Upon UV illumination, the (bPEI/APS NP)_30_ films show an orange-red fluorescence (Fig. 6D-i). The capacity of the film to detect Cu^2+^ ions is then assessed using 10 mM of CuCl_2_ solution (Fig. 6D-ii). While an orange-red fluorescence remains after the addition of water to the left portion of the stick, it is quenched upon the addition of CuCl_2_ solution to the right portion of the stick. Finally, it should be noted that the (bPEI/APS NP)_30_ LbL stick kit decomposes within 10 min in aqueous solution (Supporting Fig. 5D), supporting the use of the kit as a quick and portable means to measuring [Cu^2+^].

## Conclusion

In this study, we evaluate the potential of APS NPs for the development of two separate copper ion detection methods. The synthesized APS NPs have good PL stability against variations in pH and time. In particular, given the stability of PL intensity in low pH, the APS NPs could potentially be used in probes for acidified soil or water with minimal loss in sensitivity. As a proof of concept, we engineer a 96-well paper kit coated with APS NPs for the detection of [Cu^2+^], which has a detection range of 1 μM to 200 μM. We also demonstrate the use of APS NPs in a portable stick-based system, in which a LbL process is used for the detection ability of [Cu^2+^]. Collectively, we engineer APS NPs and demonstrate their potential as sensors for copper contamination.

## Methods

### Preparation of PS NPs

N-type, As-doped silicon wafers with a diameter of 500 mm (2 in), an <111> orientation, and resistivity of 0.001–0.01 Ω·cm were used in this study. The Si wafers were electrochemically etched in an HF–H_2_O–ethyl-alcohol (1:1:4 v/v) mixture after lateral etching, following a procedure reported elsewhere^24^,^25^. In brief, anodic etching was performed in a polycarbonate cell with dimensions of 150 × 60 × 50 mm. The silicon wafer was located between Pt wires that were meshed on parallel Teflon plates, and electrical contact was provided to the top edge of the silicon wafer with silver paste. An electrolyte was slowly pumped into the cell (Masterflex L/S Pump, Cole Parmer, USA) at a speed of 5 mL/min. The total etching time was approximately 1 hr at a constant current of 120–250 mA (Model PS300c, SolGent, Korea). Following anodic etching, the wafers were washed with copious amounts of deionized water and methanol (HPLC grade, Fisher) and dried with nitrogen gas. The dried wafers displayed an intense orange-red or yellow-blue luminescence when excited at 365 or 254 nm, respectively. The dried wafers were then sonicated for 90 min in 20 mL of ethyl alcohol. The sonicated suspensions were brownish and exhibited unique broadband PL at around 365 nm. The suspensions were centrifuged at 13,000 *g* (*g* = 9.81 m s^−2^) for 10 min to separate the PS NPs.

### Photo-Initiated and Thermo-Initiated Hydrosilylation of PS NPs

APS NP suspension with AA (10 wt% in ethanol) was first bubbled with N_2_ to remove any dissolved oxygen that could inhibit the radical-initiated reaction. Next, the PS NP suspension was placed in a photochemical reactor (Rayonet, Southern New England Ultraviolet Co., USA) equipped with 10 UV tubes (RPR-2537 Å, Southern New England Ultraviolet Co., USA) to initiate the hydrosilylation reaction; the reaction ran for 24 h at 40 °C and produced a clear suspension. To generate UDA-PS NPs, 20 mL of PS NPs were transferred to a three-necked flask containing UDA (10 wt% in ethanol) and heated at 95 °C for 16 h with continuous stirring under a nitrogen atmosphere. The turbid suspension became transparent upon hydrosilylation of the PS NPs with UDA. Both types of suspensions were centrifuged at 13,000 *g* for 10 min to separate APS NPs, after which the NPs were washed with ethanol and centrifuged three more times to ensure thorough washing. Finally, 500 μL of deionized water was added to resuspend the APS NPs. The resulting APS NP suspension displayed orange-red florescence under 365-nm excitation.

### Measurement of Quenching Efficiencies by Other Ions

10 mM Fe^2+^, Na^+^, K^+^, Mg^2+^, Mn^2+,^ Ca^2+^, and Cu^2+^ were used to observe the presence and extent of fluorescence quenching of UDA-PS NPs in water. 200 μL of UDA-PS NP solution was placed in a 96 well plate, after which 10 μL of each ion was added to each well. Endpoint fluorescence measurements were taken at a wavelength of 665 nm.

### Preparation of a Paper Kit

Sheets of Whatman cellulose filter paper No. 1 were cut into pieces measuring 128 mm × 85 mm to fit into the microplate reader. We designed a 96-well paper kit (Adobe Illustrator CS6, Adobe, USA) with 5-mm-diameter wells to accommodate 20 μL of APS NP sample in each well. The paper kit was printed with a wax printer (ColorQube 8570, Xerox, Japan), after which the printed paper was baked at 130 °C for 3 min to melt the wax ink into the paper to form a hydrophobic wall surrounding each well. A piece of Parafilm was then attached under the paper kit to prevent the leaking of any liquid from the wells. Finally, the paper kit was placed on a polypropylene plate for steady delivery to a microplate reader.

### Stacking of APS NPs on a Paper Kit

An aqueous solution of APS NPs was coated onto the paper kit by adding 20 μL droplets of the particle solution into individual wells. The paper kit was then stored in a vacuum dry oven for 1 h at 40 °C and −76 mmHg. The entire process was repeated five times, after which the wells exhibited a bright PL upon UV excitation under 356 nm.

### Preparation of PDMS-Patterned (bPEI/pSiNPs)_*n*_ Films by LbL Assembly

bPEI (*M*_W_ = ~2,000) was purchased from Sigma-Aldrich, USA. The bPEI and APS NPs were separately dissolved in ethanol to concentrations of 1 mg/mL. A curing agent and a PDMS prepolymer (SYLGARD 184 Silicone Elastomer Kit, Dow Corning, USA) were thoroughly mixed at a ratio of 1:10 v/v. The prepolymer mixture was poured onto substrates made of an overhead projector (OHP) transparency film and cured for 3 h at 100 °C in a vacuum oven. After curing, the thin PDMS replicas were peeled off the substrates, onto which LbL films were fabricated. The PDMS-patterned OHP film substrates were cleaned and functionalized by oxygen-plasma treatment (CUTE-1B, Femto Science, USA). The multilayered (bPEI/pSiNP)_*n*_ films were then prepared as follows. First, 20 μL droplets of the bPEI solution were added onto the PDMS-patterned OHP film substrate and silicon wafer and allowed to stand for 10 min to enable hydrogen bonding between the hydroxyl groups of the substrate surface and amine groups of bPEI. Subsequent washing in an ethanol stream three times eliminated any weakly-bound bPEI. 20 μL droplets of APS NP solution were then added to the bPEI-coated surface and washed with ethanol in a similar manner.

### Characterization of APS NPs

The APS NPs were characterized by TEM. TEM and high-resolution TEM (HRTEM) images were obtained with a JEM-2100F microscope (JEOL, Japan) at an acceleration voltage of 200 kV. The TEM samples were prepared by drying droplets of the NP suspension in ethanol on a 300-mesh copper grid coated with carbon film. The PL and UV absorption spectra of the APS NPs were recorded using Synergy Mx (Biotek Inc., USA). The particle size distribution and zeta potential of the APS NPs were analyzed using a dynamic light-scattering system (Zetasizer, Malvern, UK) and TEM. To calculate the quenching efficiency (Δ%) of PL, the PL standard intensity (*f*_std_) was compared to the reduced PL intensity (*f*_re_) while maintaining the same values for the excitation intensity, absorbance, and other instrumental parameters. The quenching PL intensity ratio was obtained as a percentage:


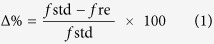


### Characterization of LbL (bPEI/pSiNPs)_*n*_ Films

The growth, thickness, and degradation rate of the films were evaluated using a profilometer (Dektak 150). FE-SEM images were obtained with a scanning electron microscope (Carl Zeiss, Germany).

### Measurement of Quenching Efficiency

The quenching efficiency of copper ions towards APS NPs was assessed by adding 200 μL of APS NP solution into 96 wells, after which copper ion solutions were added to select wells that had comparable initial levels of PL intensity. Similarly, the quenching efficiency of copper ions towards UDA-PS NPs on a paper-based kit was measured by adding 10 μL of a standard copper ion solution to a completely dried paper kit.

### Supporting Information

Supporting information contains additional data on the characteristics of the APS NPs including quenching selectivity, quantum yield, quenching efficiency, fluorescence stability in biological fluids, and thicknesses of nanofilms containing the NPs.

## Additional Information

**How to cite this article**: Hwang, J. *et al*. Sensitive detection of copper ions via ion-responsive fluorescence quenching of engineered porous silicon nanoparticles. *Sci. Rep.* **6**, 35565; doi: 10.1038/srep35565 (2016).

## Supplementary Material

Supplementary Information

## Figures and Tables

**Figure 1:**
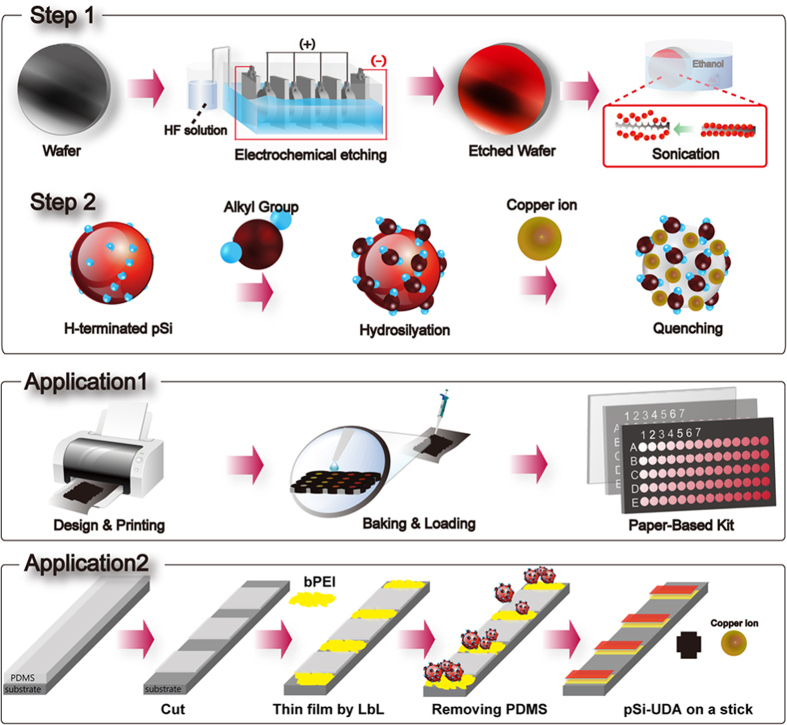
Schematic drawing detailing the preparation and application of alkyl-terminated porous silicon nanoparticles (APS NPs). Step 1: Silicon-wafer etching in an electrochemical bath and procurement of Si particles via sonication. Step 2: UV-illumination-assisted hydrosilylation on the hydrogen-rich surface of PS NPs for the generation of APS NPs, which exhibit PL quenching in the presence of copper ions. Application 1: Preparation of a 96-well paper kit, which consists of APS NP deposition onto wax printed paper. Application 2: Preparation of a portable stick kit, fabricated via layer-by-layer (LbL) assembly onto a polydimethylsiloxane (PDMS)-patterned flexible overhead projector (OHP)-film substrate.

**Table 1:** Detection of [Cu^2+^] in tap water via APS NP-based paper kit.

Water	0	0	0	0
Cu^2+^ conc. (μM)	0	5	25	50
Average Δ%	0.03	10.57	26.52	55
Standard deviation	0.01	2.38	1.04	2.21
ICP (mg/L)	0.01	0.18	1.25	2.5

The absolute amount of copper ions in the sample is obtained by ICP mass spectrometry.
