# Supplementary material for: Sensitive detection of copper ions via ion-responsive fluorescence quenching of engineered porous silicon nanoparticles
Source: Sci Rep. 2016 Oct 18;6:35565. doi: 10.1038/srep35565 (PMC5067703; doi:10.1038/srep35565)
Supplement: Supplementary Information [file srep35565-s1.doc]

**Supporting Information:**

Sensitive detection of copper ions via ion-responsive fluorescence quenching of engineered porous silicon nanoparticles

Jangsun Hwang,^a,†^ Mintai P. Hwang,^b,†^ Moonhyun Choi,^c^ Youngmin Seo,^a^ Yeonho Jo,^a^ Jaewoo Son,^a^ Jinkee Hong,^c,*^ and Jonghoon Choi^a,*^

^a^School of Integrative Engineering, Chung-Ang University, Seoul 06974, Republic of Korea

^b^Department of Bioengineering, Swanson School of Engineering, University of Pittsburgh, Pittsburgh, PA 15261 USA

^c^School of Chemical Engineering & Materials Science, Chung-Ang University, Seoul 06974, Republic of Korea


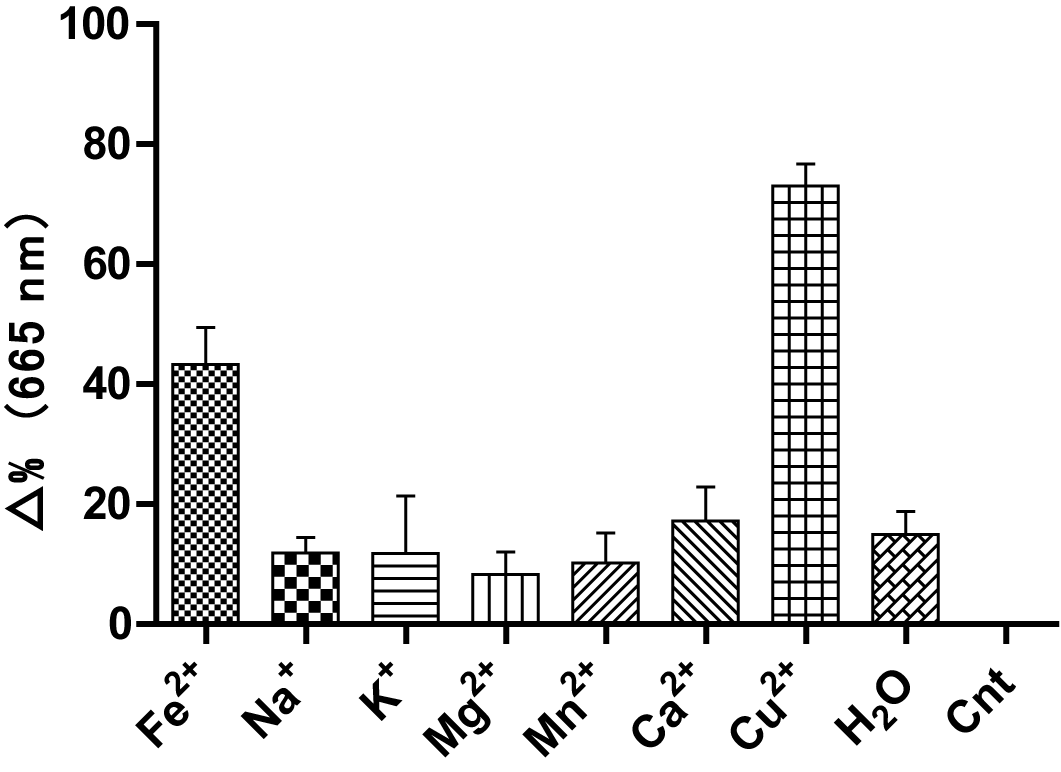


**Figure S1.** UDA-PS NP quenching efficiency of several ions. 10 μL of 10 mM ion solutions are added to UDA-PS NPs and measured for fluorescence intensity at 360 nm (Cnt: untreated).


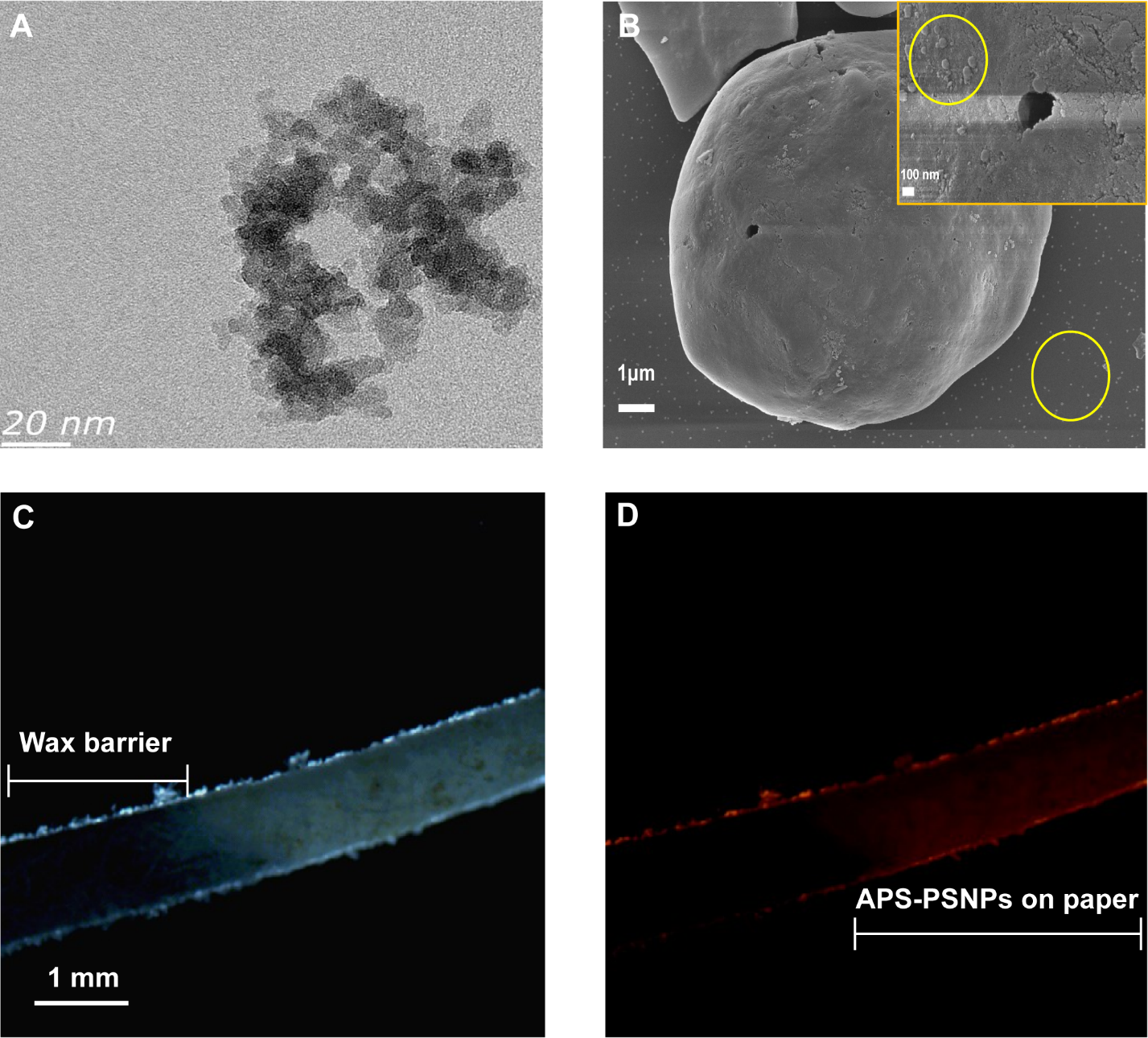


**Figure S2.** (A) Typical aggregation behavior of freshly etched porous silicon nanoparticles. (B) SEM image of bare PS NPs (PS NPs were located in yellow circles). (C) Wax printed paper kit after baking process (wax barrier is shown in black). (D) APS-PS NPs stacked on a paper kit are illuminated under UV excitation.

**Figure S3.** Quantum yield measurement of APS NPs. The quantum yield (Ф) of UDA-PSNPs is measured by comparing its PL intensities to those of Rhodamine. The PL intensity for Rhodamine is integrated between 560 and 700 nm; the quantum yield (Ф) is 0.83. The calculated quantum yield of UDA-PSNPs is 0.11.


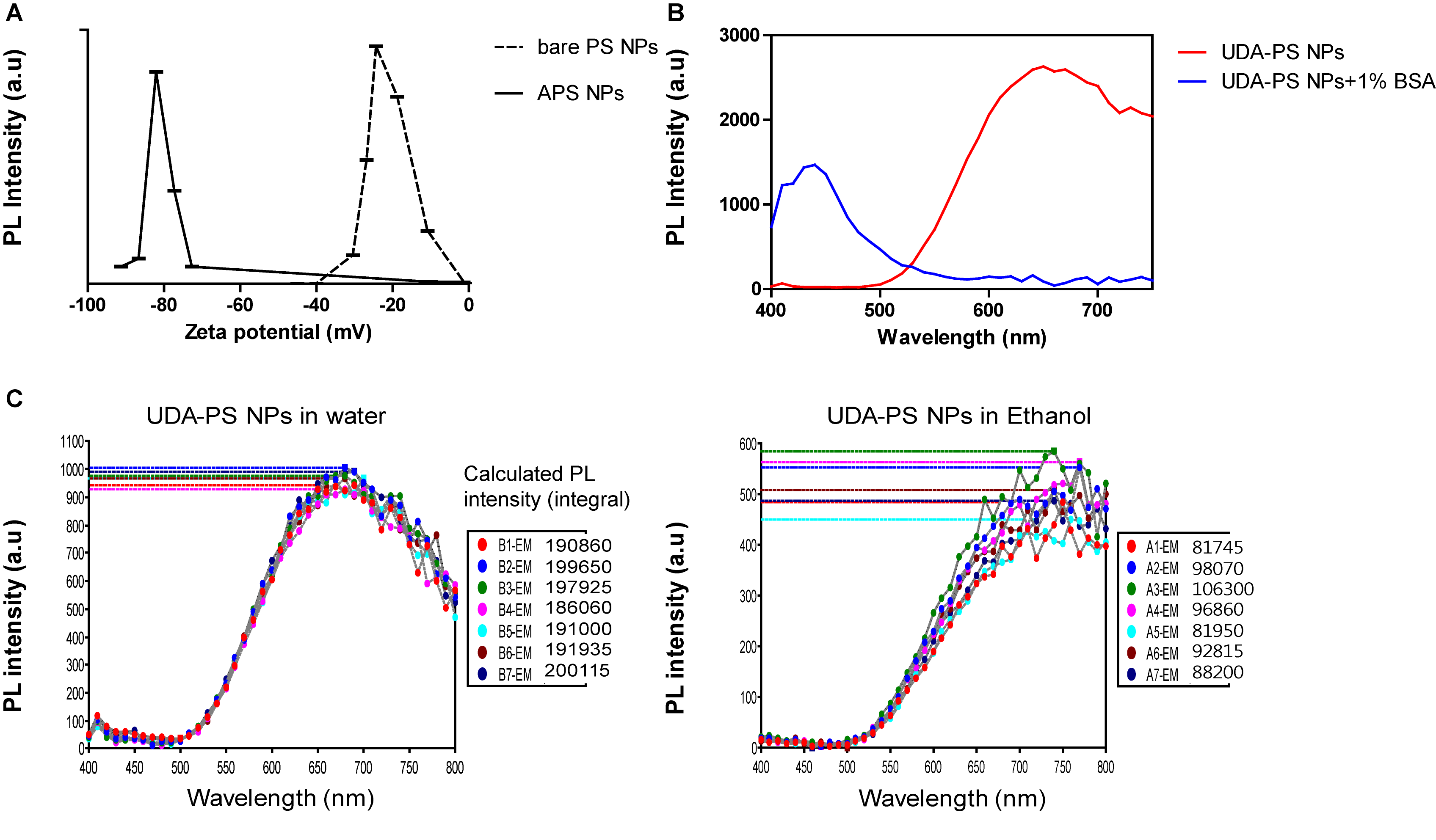


**Figure S4.** (A) Zeta potential of APS-PS NPs and bare-PS NPs in water. (B) PL intensity peak shift in the presence of a biomolecule (1% BSA, blue shifting). (C) PL intensity of UDA-PS NPs in water are higher than those in ethanol.


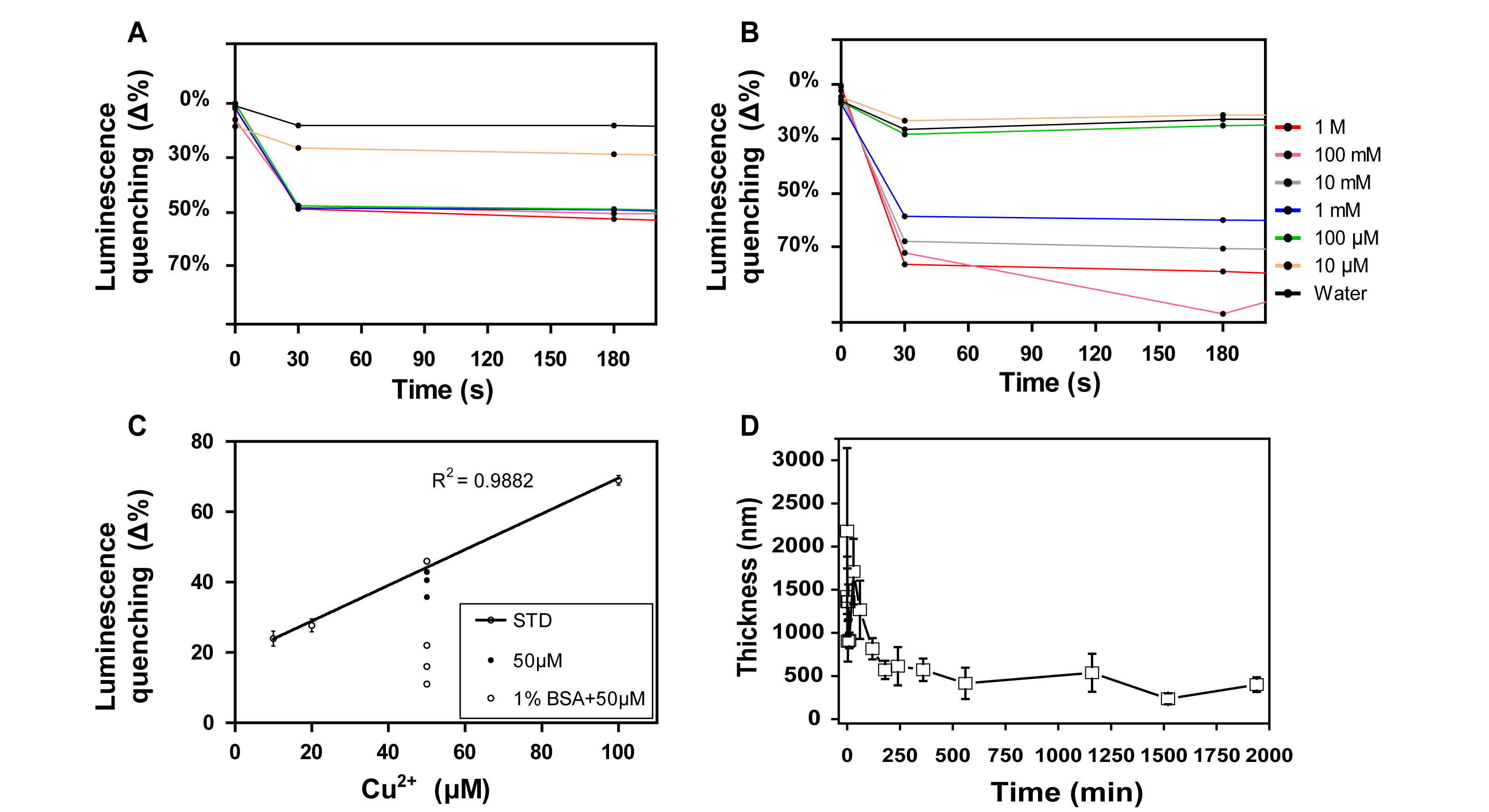


**Figure S5.** Time-dependent fluorescence quenching efficiencies of (A) UDA-PS NPs and (B) AA-PS NPs dispersed in solutions containing different concentrations of CuCl_2_. (C) Copper ion measurement under physiological conditions (1% BSA). Autofluorescence from BSA shifted the emission to blue; physical interaction with nanoparticles also deteriorated the measured fluorescence. (D) Degradation rate of (bPEI/A-pSiNP)_30_ LbL film is assessed by dipping a flexible stick into PBS (pH 7.4).
